# Biochemical Issues in Estimation of Cytosolic Free NAD/NADH Ratio

**DOI:** 10.1371/journal.pone.0034525

**Published:** 2012-05-03

**Authors:** Feifei Sun, Chunyan Dai, Jiansheng Xie, Xun Hu

**Affiliations:** Key Laboratory for Cancer Intervention and Prevention, Zhejiang Provincial Key Laboratory of Molecular Biology in Medical Sciences, China National Ministry of Education, Cancer Institute, School of Medicine, Zhejiang University, The Second Affiliated Hospital, Hangzhou, Zhejiang, China; Sun Yat-sen University Cancer Center, China

## Abstract

Cytosolic free NAD/NADH ratio is fundamentally important in maintaining cellular redox homeostasis but current techniques cannot distinguish between protein-bound and free NAD/NADH. Williamson et al reported a method to estimate this ratio by cytosolic lactate/pyruvate (L/P) based on the principle of chemical equilibrium. Numerous studies used L/P ratio to estimate the cytosolic free NAD/NADH ratio by assuming that the conversion in cells was at near-equilibrium but not verifying how near it was. In addition, it seems accepted that cytosolic free NAD/NADH ratio was a dependent variable responding to the change of L/P ratio. In this study, we show (1) that the change of lactate/glucose (percentage of glucose that converts to lactate by cells) and L/P ratio could measure the status of conversion between pyruvate + NADH and lactate + NAD that tends to or gets away from equilibrium; (2) that cytosolic free NAD/NADH could be accurately estimated by L/P only when the conversion is at or very close to equilibrium otherwise a calculation error by one order of magnitude could be introduced; (3) that cytosolic free NAD/NADH is stable and L/P is highly labile, that the highly labile L/P is crucial to maintain the homeostasis of NAD/NADH; (4) that cytosolic free NAD/NADH is dependent on oxygen levels. Our study resolved the key issues regarding accurate estimation of cytosolic free NAD/NADH ratio and the relationship between NAD/NADH and L/P.

## Introduction

Cytosolic free NAD/NADH ratio plays a very important role in maintaining cellular redox homeostasis and it could be generally considered as a cellular metabolic readout [1], [2]. Given the fact that over 700 oxidoreductive enzymes use NAD or NADH as cofactors, the cytosolic free NAD/NADH ratio is globally involved in cellular biochemical processes. Therefore, a change of cytosolic free NAD/NADH could infer a metabolic alteration and is closely linked to physiological or pathological states [3]–[24].

NAD and NADH in cells exist in protein-bound and free forms. Current techniques can only measure total NAD and total NADH [2], [25]–[29] but could not distinguish between protein-bound and free form. Since it is the free form of NAD/NADH that regulates cellular redox potential, measurement of total NAD/NADH virtually tells no information of in vivo redox state. Williamson et al developed a method to estimate the cytosolic free NAD/NADH ratio based on the principle of chemical equilibrium [30]. When the conversion between pyruvate + NADH and lactate + NAD is at equilibrium, cytosolic free NAD/NADH ratio could be calculated by L/P ratio according to the equation of chemical equilibrium. The procedure to determine the ratio involves 3 steps: first, assume the intracellular conversion between pyruvate + NADH and lactate + NAD is at equilibrium or near-equilibrium; second, quantitatively determine intracellular pyruvate and lactate; finally, calculate the ratio according to the equation as described above.

How to correctly apply this method to estimate cytosolic free NAD/NADH remains a problematic issue. Many studies ([3]–[24], [31]–[41]) seem to have the measurement problem which apparently persists and has not been recognized at all. The reason that leads to the incorrect measurement is owing to the misuse of the equation of chemical equilibrium. In the cited studies, the cytosolic NAD/NADH ratio was calculated by lactate/pyruvate based the equation of chemical equilibrium, simply assuming that the conversion in cells was at near equilibrium without verifying how near it was. This is rationally incorrect, because the mass action ratio (Q) at near-equilibrium could differ from the Keq at equilibrium by 1 or 2 orders of magnitude ([42]). Hence, cytosolic NAD/NADH estimated as such could be deviated from the true values by 1 or 2 orders of magnitude. This has been a blind spot that misleads the estimation ever since. Another equally important issue is the relationship between NAD/NADH and L/P, which is not clear. The cytosolic free NAD/NADH ratio seems to be regarded as a variable that is dependent on the cytosolic L/P ratio, hence a change of L/P under different physiological and patholological conditions represents a corresponding change of NAD/NADH [10]–[22], [31]–[36]. However, it should be borne in mind that NAD/NADH is not necessarily a dependent variable that responds to the change of cytosolic L/P ratio. The fact that cytosolic free NAD/NADH ratio is involved in many biochemical reactions argues strongly against its dependence on a single reaction in cells. We reiterate, however, that we do not at all intend to criticize the previous studies, but that it is very important to resolve the issue regarding how to correctly apply this method in order to accurately estimate the ratio. Lin & Guarente [2] in their review article summarized the reported values of cytosolic free NAD/NADH in various tissues or cells from different species, which varied markedly from 0.1 to 644, hence they pointed out that ‘to understand the role of NAD as a metabolic regulator, it is therefore very important to investigate which reported number for the NAD:NADH ratio represents the real situation in cells’.

In this study, we sought to resolve the following issues. How is the conversion at equilibrium or at near-equilibrium in cells defined? What significant calculation error might be introduced when the method is applied to estimate cytosolic free NAD/NADH without verifying the equilibrium status of the conversion? Is the cytosolic free NAD/NADH dependent on cytosolic L/P ratio? Is this ratio a dependent variable responding to oxygen level?

Unless otherwise indicated, the word conversion indicates the conversion between lactate + NAD and pyruvate + NADH, and the NAD/NADH ratio indicates cytosolic free NAD/NADH ratio.

## Results and Discussion

### Intracellular Lactate Concentration and L/P Ratio are Highly Labile

**Figure 1:**
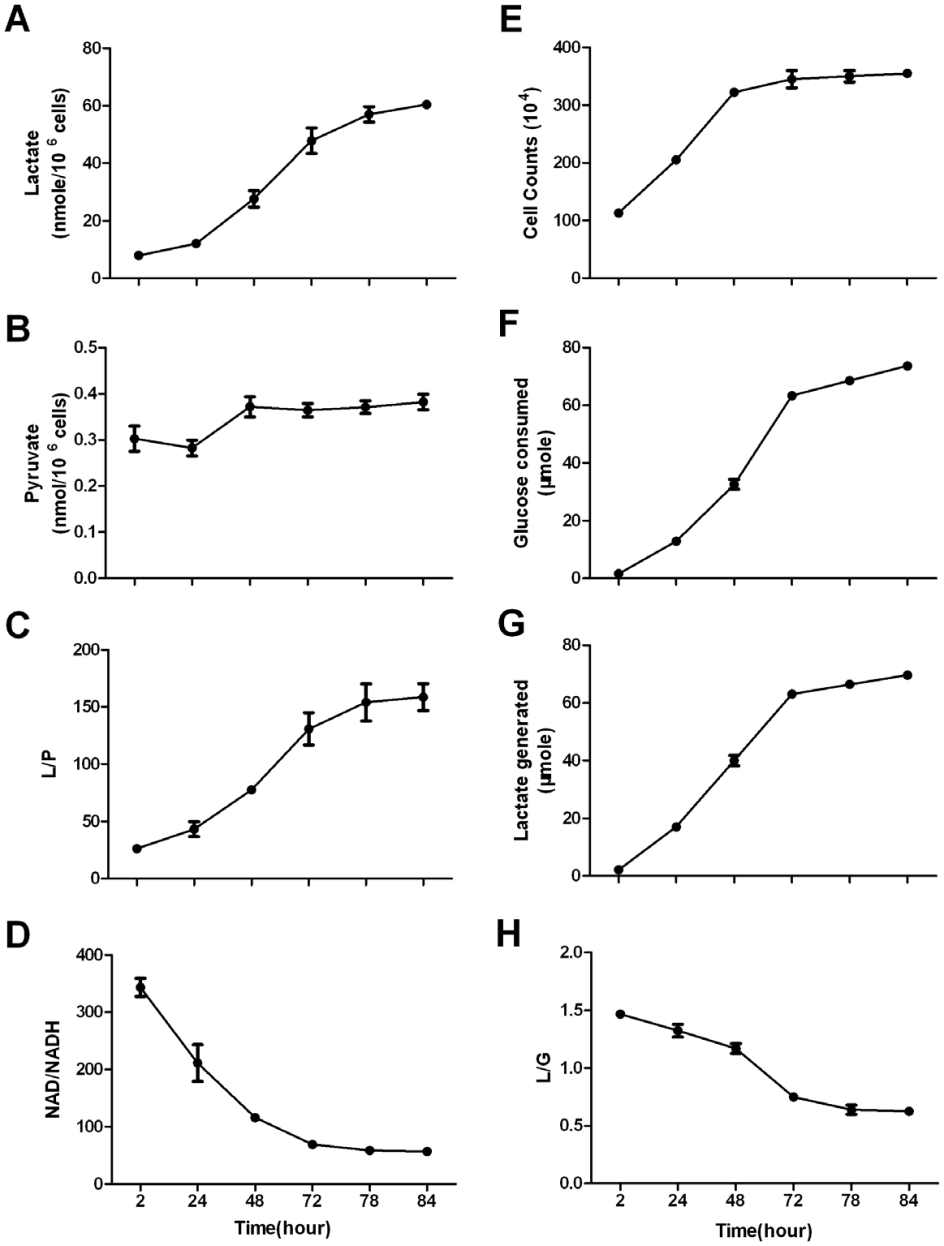
Intracellular lactate concentration and L/P ratio are highly labile in Bcap-37 cells. Bcap-37 cells were incubated in complete RPMI-1640 medium containing 12 mM glucose and supplemented with 6 mM glucose every 24 hours. At the indicated time point, intracellular lactate and pyruvate, glucose consumption and lactate generation by Bcap-37 cells, and cell growth were monitored. (A) Intracellular lactate; (B) Intracellular pyruvate; (C) Intracellular L/P ratio; (D) Cytosolic free NAD/NADH ratio erroneously estimated from the corresponding L/P ratio; (E) Cell proliferation curves; (F) Glucose consumption; (G) Lactate generation; (H) L/G ratio (the generated lactate divided by the consumed glucose between 2 time points). Data are mean±SD. Data were confirmed by 2 independent experiments.

Human breast cancer cell line Bcap-37 cells were incubated in medium with sufficient supply of glucose. At indicated intervals, intracellular pyruvate and lactate were measured. It was noted that intracellular lactate concentration changed drastically throughout the incubation time (Figure 1A), whereas pyruvate concentration kept relatively constant (Figure 1B). Thus, intracellular lactate concentration and L/P ratio were highly labile (Figure 1C). L/P ratios at 2- and 84-hour points were 26.2±1.2 and 158.6±11.8, respectively. If the equation of the chemical equilibrium was used to estimate NAD/NADH ratio regardless of the equilibrium status of the conversion, the estimated NAD/NADH ratios in Bcap-37 could vary from 343.3±15.7 to 57±4 (Figure 1D).

Apparently, such estimated NAD/NADH ratios were highly problematic. In the previous studies, it was envisaged that NAD/NADH ratio was relatively constant under physiological condition [10]–[22], [31]–[36]. In cells, there are over 700 oxidoreductases that use NAD/NADH as cofactors to catalyze numerous biochemical reactions involving endogenous or exogenous reactants. In cells, except the exergonic reactions that are far from equilibrium, most reactions are reversible and at near-equilibrium. At near-equilibrium, Q and Keq often differ by one order of magnitude of each other, there is a small difference between forward and reverse rates of reaction, so that small change of substrate/product ratio can result in a large change of reaction rate and even can change the direction of the net flow [42]. Because NAD and NADH are reciprocal substrate and product in these reactions, the change of the ratio directly interferes with the rate or even the direction of numerous oxidoreductive reactions. If NAD/NADH ratio was highly unstable, the rate and the direction of these reactions would be elusive, and cellular redox homeostasis would be easily disturbed. Thus, NAD/NADH ratio should be relatively constant in order for the physiological processes to proceed.

The change of the intracellular lactate was simply a result of lactate production by Bcap-37 cells. Bcap-37 cells are highly glycolytic and they can convert most of the incoming glucose to lactate, as manifested by the glucose consumption and lactate generation accompanying their growth and proliferation (Figure 1E–G). This explains the positive correlation between incubation time and intracellular lactate concentration.

### L/G and L/P Could Measure the Conversion Status between Lactate + NAD and Pyruvate + NADH that Tends to or Gets Away from Equilibrium

It was noted that L/G changed significantly throughout the incubation (Figure 1H). L/G ratio indicates percentage of incoming glucose that converts to lactate. One molecule of glucose via complete aerobic glycolysis can yield 2 molecules of lactate. Therefore, L/G ratio is ranging between 2 – 0, reflecting 100 – 0% incoming glucose (or pyruvate and NADH generated from glycolysis) converting to lactate. The L/G value also reflects the status of the conversion between pyruvate/NADH and lactate/NAD, the smaller the value, the closer the conversion is to the equilibrium, and vice versa. At initial culture, the L/G ratio is 1.47 (Figure 1H), indicating that ∼75% of pyruvate/NADH generated from glycolysis converted to lactate/NAD. Extending incubation time led to lactate accumulation, and L/G ratio gradually decreased to 0.63±0.04, indicating that only ∼30% of pyruvate/NADH converted to lactate/NAD, hence the conversion from pyruvate to lactate was approaching equilibrium, suggesting that the estimated NAD/NADH ratio at 84-hour incubation point was closest to the true value in cells.

Under regular culture, the conversion was at near-equilibrium state, as demonstrated by the change of intracellular L/P (Figure 1C) and L/G (Figure 1H) throughout the entire incubation. The intracellular L/P ratio only increased by 6 folds, whereas the L/G ratio was significantly reduced from 1.47±0.06 to 0.63±0.04, indicating the conversion was approaching equilibrium. If the conversion was far from equilibrium, 6-fold change of product/substrate ratio would not significantly affect the conversion state [42]. Thus, our data are fully agreeable with the generally accepted perception that this conversion in cells is at near equilibrium [10]–[22], [31]–[36].

Hence, L/G and L/P could measure the conversion status between NAD + lactate and pyruvate + NADH that tends to or gets away from equilibrium.

Similar results were obtained by experimentations on other cancer cell lines (Figure S1 and S2).

### Defining the Equilibrium Status of the Conversion is Essential to Estimate NAD/NADH Using L/P

How to define the equilibrium status of the conversion is a key issue to correctly estimate NAD/NADH. The equilibrium status of the conversion in cells could be defined by its rate. The net flow of the conversion depends on the intracellular Q. When the Q is smaller than the Keq, reaction proceeds to the direction of lactate formation; when the Q is equal to the Keq, the forward and reverse rates are equal, there is neither gain nor loss of lactate; when the Q is bigger than the Keq, the reaction proceeds to pyruvate formation. Based on this principle, we can verify whether this reaction in cells is at equilibrium or not.

Most cancer cells have very high glycolytic rates that result in excessive generation of pyruvate and NADH [43]–[45], which are beyond the capacity of pyruvate dehydrogenase and NADH mitochondrial shuttle [46]. As a result, excessive pyruvate and NADH are instantly converted to lactate and NAD by lactate dehydrogenase (LDH). Lactate is then disposed by cells via monocarboxylate transporters (MCT) [47]. Therefore, most cancer cells in culture produce quantity of lactate, reflecting that the net flow of the intracellular conversion is from pyruvate to lactate. On the other hand, considering the conversion is at near-equilibrium in cells, lactate accumulation would eventually bring the conversion to equilibrium. In order to test this hypothesis, we incubated cells in culture medium supplemented with serial concentrations of lactate. Cells would uptake lactate via MCT [47]. When intracellular lactate concentration increases to a certain level, the Q would be equal to Keq, leading to the conversion at equilibrium.

**Figure 2:**
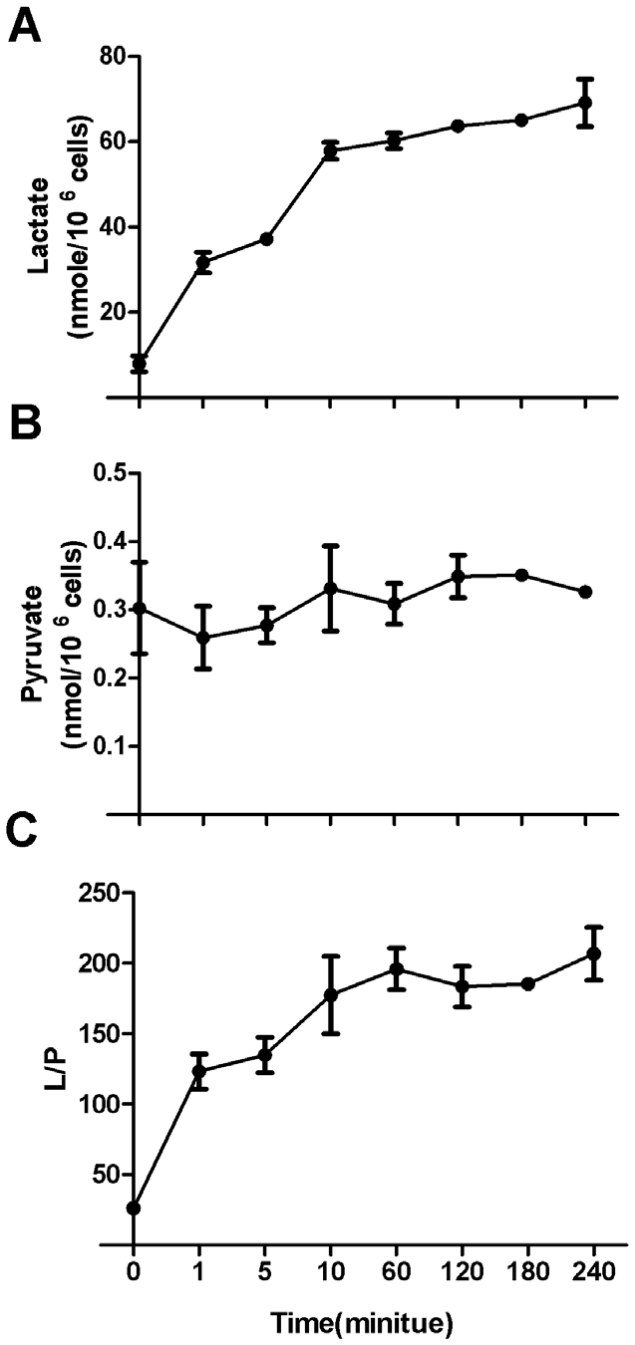
The effect of extracellular lactate concentration on intracellular lactate concentration and intracellular L/P ratio in Bcap-37 cells. Bcap-37 cells were incubated in complete RPMI-1640 medium containing 12 mM glucose supplemented with 20 mM lactate. At the indicated time point, intracellular lactate and pyruvate were measured. (A) Intracellular lactate; (B) Intracellular pyruvate; (C) Intracellular L/P ratio. Data are mean ± SD. Data were confirmed by 2 independent experiments.

In order to see the effect of extracellular lactate on intracellular L/P ratio, we incubated Bcap-37 cells in culture medium containing 20 mM lactate and determined time-dependent lactate uptake. The steady-state lactate accumulation was attained in 10 minutes (Figure 2A), indicating a fast lactate uptake by Bcap-37 cells or a fast equilibrium of lactate concentration across plasma membrane. On the other hand, intracellular pyruvate concentration was not significantly changed (Figure 2B). As a result, L/P ratio throughout entire incubation increased by about 8 folds. Given the near-equilibrium status of the conversion in cells with Q value that is smaller but often within one order of magnitude less than Keq, this 8-fold increase of L/P ratio would likely lead the conversion to equilibrium.

**Figure 3:**
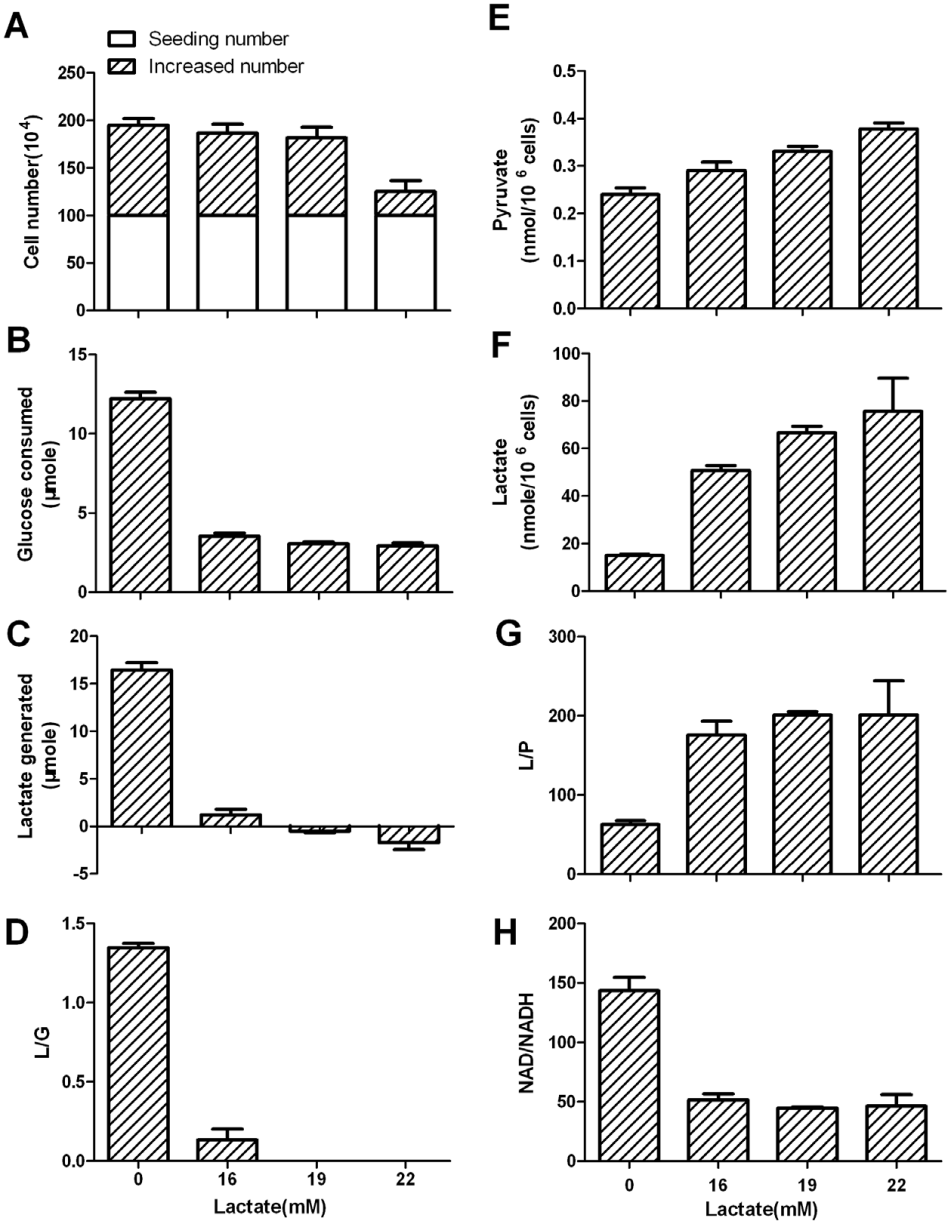
Cytosolic free NAD/NADH ratio estimated at the conversion at equilibrium in Bcap-37 cells. Bcap-37 cells were incubated in complete RPMI-1640 medium containing 12 mM glucose supplemented with or without lactate (x axis). After 24-hour incubation, glucose consumption and lactate generation by Bcap-37 cells, cell growth, and intracellular lactate and pyruvate were measured. (A) Cell proliferation; (B) Glucose consumption; (C) Lactate generation; (D) L/G ratio; (E) Intracellular pyruvate; (F) Intracellular lactate; (G) Intracellular L/P ratio; (H) Cytosolic free NAD/NADH ratio estimated from the corresponding L/P ratio(see corresponding text). Data are mean±SD. Data were confirmed by 3 independent experiments.

In order to test whether the increase of L/P ratio could affect the equilibrium state, we measured glucose consumption and lactate generation by Bcap-37 cells that were incubated in medium containing exogenous lactate (Figure 3A–D). In the absence of exogenous lactate, cells converted quantity glucose to lactate with a high L/G ratio (Figure 3D), indicating that the net flow is from pyruvate to lactate. In the presence of exogenous 16 mM lactate, the L/G ratio was dramatically reduced to 0.2±0.03, indicating that the conversion was approaching equilibrium. In the presence of 19 mM lactate, cells produced negligible lactate and the L/G ratio was close to zero, indicating that the conversion was at or very close to equilibrium. In the presence of 22 mM lactate, cells consumed lactate, indicating that the direction of the conversion was reversed. We then measured the intracellular concentration of lactate and pyruvate, and L/P ratios in cells cultured under above conditions (Figure 3E–G). If the equilibrium state was taken into consideration, the value of NAD/NADH ratio was around 45 (Figure 3H). Otherwise, the values of NAD/NADH ratio could vary from 143.6±11.1 to 46±9.7 (Figure 3H). In principle, only when the conversion is at equilibrium, it is appropriate to use the equation ([NAD]/[NADH] = Keq×[pyruvate]/[lactate], where Keq = 9000). It is noted that when the conversion is very close to equilibrium under culture conditions of 16, 19, or 22 mM lactate, the estimated NAD/NADH values are similar to each other without significant difference. It is also noticed that NAD/NADH ratio estimated from L/P ratio at 84-hour point (Figure 1D) is close to this value. Hence, NAD/NADH ratio could be correctly estimated by L/P when the conversion is at equilibrium or very near to equilibrium.

It was noted that glucose consumption was significantly reduced in cells cultured in the presence of 19 mM exogenous lactate, but cell growth rate did not significantly slow down, in comparison to the cells cultured in the absence of exogenous lactate (Figure 3A). This reflects different efficiency of glucose utilization. In the absence of exogenous lactate, most of the incoming glucose was converted to lactate as a metabolic waste, resulting in a low efficiency of glucose utilization. In the presence of 19 mM lactate, glucose was not wasted, so that the efficiency of glucose utilization is higher.

Similar results were obtained by experimentations on other cancer cell lines (Figure S3 and S4). Our study demonstrated that NAD/NADH ratio could be accurately estimated.

### Relationship between NAD/NADH and L/P

We propose here that the highly labile L/P ratio is crucial to maintain the stability of NAD/NADH ratio. Under physiological conditions, cellular catabolism could produce more NADH and pyruvate than required [18], [20], [43]–[45]. If NADH could not be instantly recycled back to NAD, the ratio of NAD/NADH would be dramatically altered, the altered NAD/NADH ratio in turn would affect numerous oxidoredutive reactions. LDH catalyzed conversion can instantly recycle excessive NADH back to NAD and thus plays a very important role in maintaining a relatively constant NAD/NADH ratio. In line with the general biochemical principles, our data (Figure 3G, Figure S3G and S4G) also demonstrate that L/P ratio is increasing or decreasing in response to the conversion status approaching equilibrium with net flow from pyruvate to lactate or reverse. Lactate is the major variable for the L/P ratio. Cancer cells have abnormally high glycolytic rate that generates excessive pyruvate and NADH, which are instantly converted to lactate and NAD by LDH, because LDH catalyzed rate is about 2 orders of magnitude higher than the glycolytic rate (data not shown). In this conversion, since NADH is recycled back to NAD, lactate is the only net gain, so that lactate concentration and L/P are highly labile and NAD/NADH is relatively constant.

### Ignoring the Equilibrium State Could Introduce a Calculation Error by One Order of Magnitude

Simply using L/P ratio to estimate NAD/NADH ratio irrespective of equilibrium state would introduce a significant calculation error, e.g., the minimal value (Bcap-37 cells incubated in culture medium without supplementing lactate at 2-hour incubation point, Figure 1D) and the maximal value (Bcap-37 cells incubated in culture medium supplementing 22 mM lactate at 24-hour incubation point, Figure 3H) of L/P ratios were 26.2±1.2 and 201.1±4.4, respectively, so that the value of NAD/NADH calculated could vary for 8.3 folds.

### NAD/NADH is a Dependent Variable Responding to Oxygen Level

Oxygen level is the fundamental determinant of the oxidoreductive potential. Decrease of oxygen level reduces the oxidative potential and increases the reductive potential, and vice versa. Presumably, NAD/NADH is a dependent variable responding to oxygen levels.

Previously, NAD/NADH ratio under hypoxia and normoxia was simply estimated by the L/P ratio, without considering the equilibrium state of the conversion [10]–[24], [37]–[41]. Since cellular glycolysis rate is much higher under hypoxia than normoxia, the cellular lactate concentration and L/P ratio are naturally different under different oxygen levels, hence the observed change of L/P ratio principally do not represent a corresponding change of NAD/NADH ratio associated to oxygen level, unless the equilibrium status of the conversion is defined.

**Figure 4:**
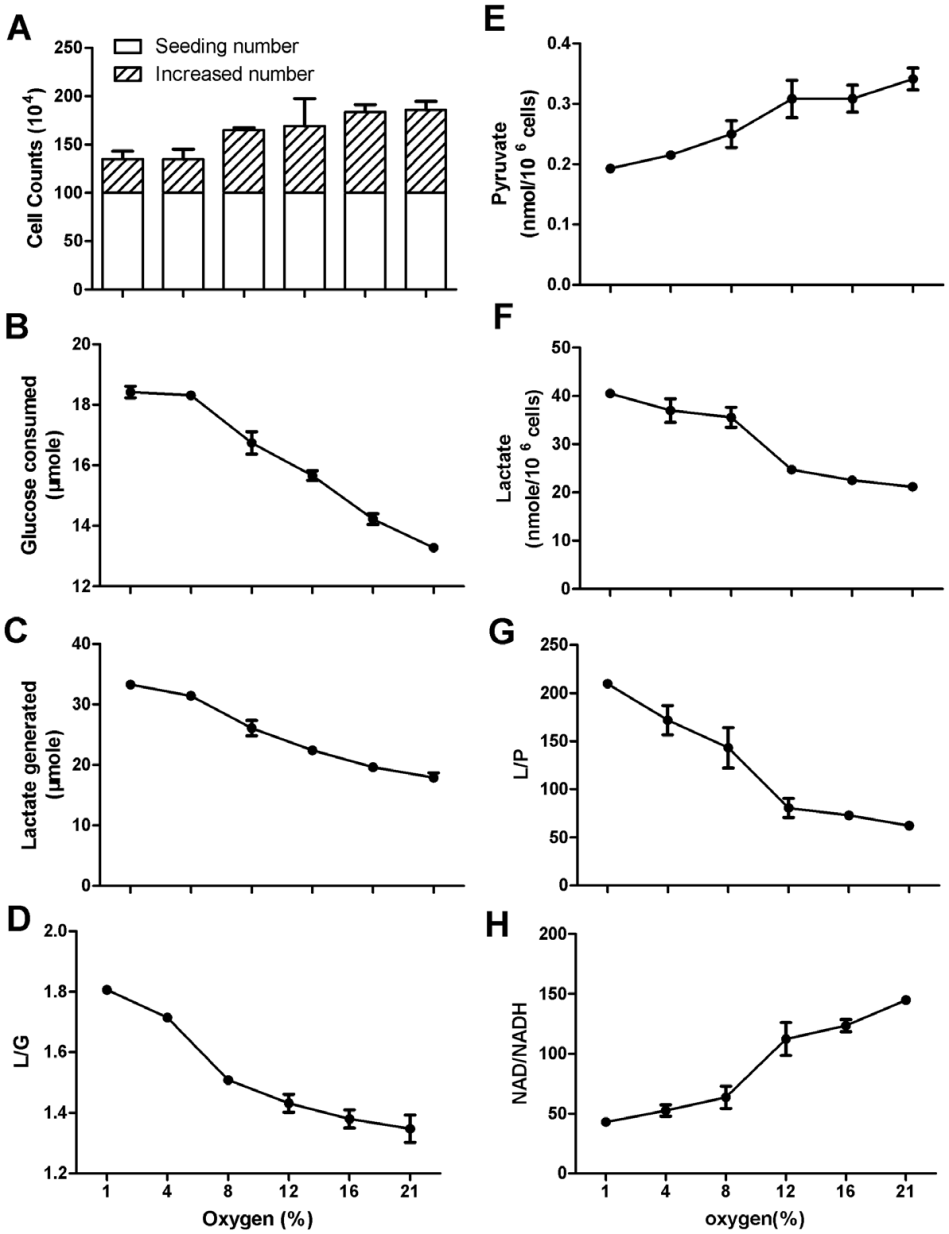
The effect of oxygen levels on intracellular concentration of lactate and pyruvate in Hela cells. Hela cells were incubated in complete RPMI-1640 medium containing 12 mM glucose. After 24-hour incubation, glucose consumption and lactate generation by Hela cells, cell growth, and intracellular lactate and pyruvate were measured. (A) Cell proliferation; (B) Glucose consumption; (C) Lactate generation; (D) L/G ratio; (E) Intracellular pyruvate; (F) Intracellular lactate; (G) Intracellular L/P ratio; (H) Cytosolic free NAD/NADH ratio erroneously estimated from the corresponding L/P ratio. Data are mean±SD. Data were confirmed by 2 independent experiments.

NAD/NADH was a dependent variable responding to oxygen level. When Hela cells were cultured without supplementing exogenous lactate and incubated under oxygen level from 21% to 1%, cell growth rate (Figure 4A) was positively correlated with, but the rates of glucose consumption (Figure 4B), lactate generation (Figure 4C), and L/G ratio (Figure 4D) were inversely correlated with the oxygen level. L/G ratios were 1.81 and 1.35 under 1% and 21% oxygen (Figure 4D), indicating that the lower the oxygen, the farther the conversion was away from equilibrium. Accordingly, the intracellular L/P ratio was inversely correlated with oxygen level, owing to decrease of pyruvate and increase of lactate (Figure 4E–G). If L/P ratio and the equation of the chemical equilibrium were used to estimate the NAD/NADH ratio, the values of NAD/NADH ratio under 21–1% oxygen could vary from 145 to 43 (Figure 4H). Such estimation could be erroneous, because the equilibrium status of the conversion was not taken into consideration, as described in the preceding sections.

**Figure 5:**
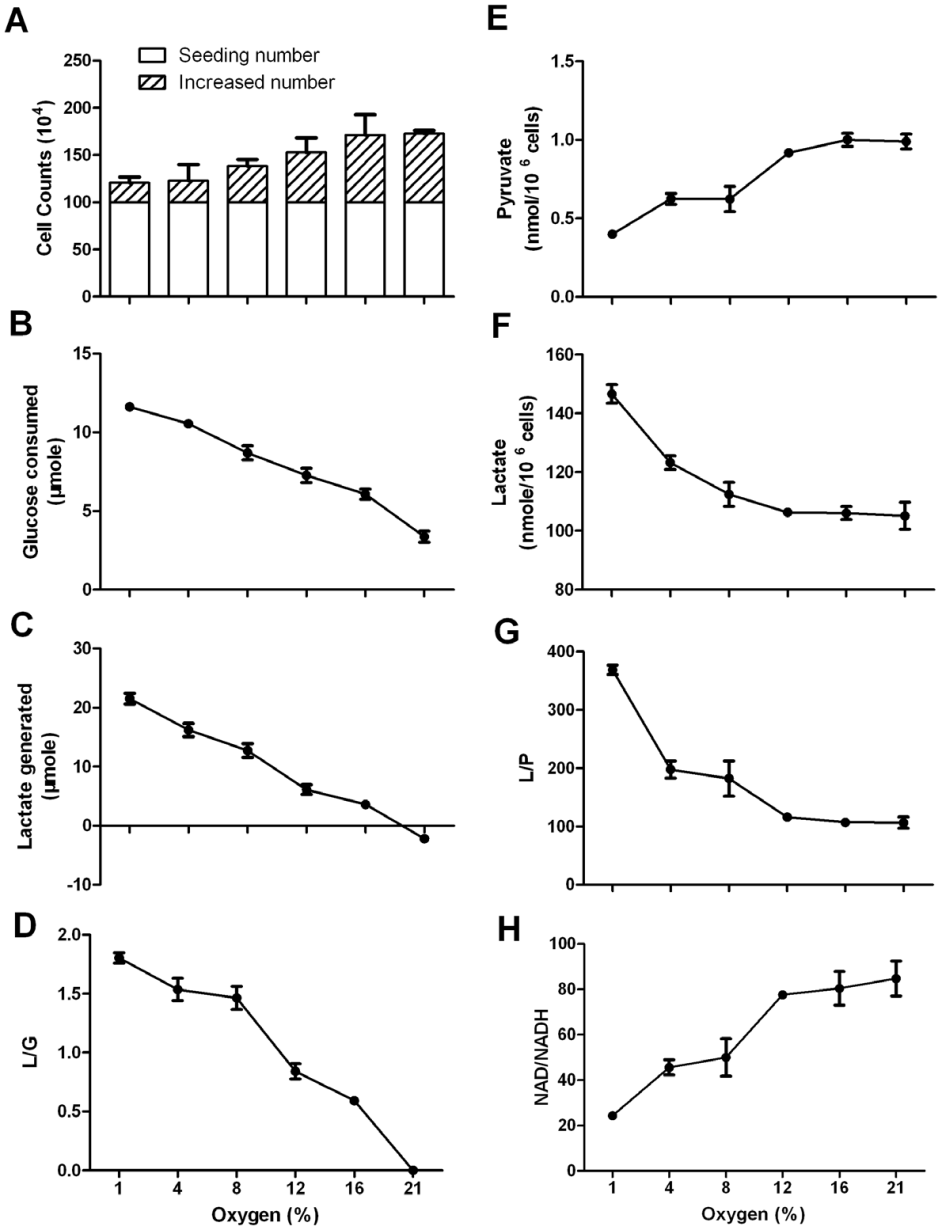
The effect of oxygen levels on cytosolic free NAD/NADH ratios in Hela cells. Hela cells were incubated in complete RPMI-1640 medium containing 12 mM glucose supplemented with 20 mM lactate under oxygen levels between 21% to 1%. After 24-hour incubation, glucose consumption and lactate generation by Hela cells, cell growth, and intracellular lactate and pyruvate were measured. (A) Cell proliferation; (B) Glucose consumption; (C) Lactate generation; (D) L/G ratio; (E) Intracellular pyruvate; (F) Intracellular lactate; (G) Intracellular L/P ratio; (H) Cytosolic free NAD/NADH ratio estimated from the corresponding L/P ratio (see description in text). Data are mean±SD. Data were confirmed by 2 independent experiments.

In order to correctly estimate the NAD/NADH ratio under different oxygen levels, we must first determine the equilibrium status of the conversion under different oxygen levels. We incubated cells in medium supplemented with 20 mM lactate under oxygen level from 21% to 1%, and monitored cell growth, glucose consumption, lactate generation, and L/G ratio (Figure 5A–D). Under 21% oxygen, there was no generation of lactate but a little consumption of exogenous lactate (Figure 5C), indicating that the Q was a little bigger than the Keq and the net flow of the conversion was from lactate to pyruvate. With the decrease of oxygen level, lactate generation was increasing (Figure 5C), indicating the net flow of the conversion was from pyruvate to lactate. The lower the oxygen, the farther the conversion was away from equilibrium, as manifested by enhanced glucose consumption, lactate generation, and increasing L/G ratio (Figure 5B–D). Intracellularly, whereas lactate concentration was inversely correlated with oxygen level, pyruvate concentration was on the opposite, resulting in an inverse correlation of L/P ratio with the oxygen level (Figure 5E–G). When oxygen level was 1%, despite that intracellular L/P was increased to 369, the conversion was far from equilibrium, as reflected by glucose consumption, lactate generation, and the L/G ratio (Figure 5B–D). Under 1% oxygen, the L/G ratio was 1.8, which was virtually the same as that in culture without exogenous lactate, indicating the conversion was not close to equilibrium. Thus, the NAD/NADH ratio estimated under 1% oxygen should be far smaller than 24 (Figure 5H), in contrast to 88 under normoxia (figure S3H). It should be also pointed out that the NAD/NADH ratios in cells under oxygen levels (1–16%) in Figure 5H were all overestimated to a varying degree and thus did not represent the true values, given the fact that the conversion was not at equilibrium. We were not able to accurately measure the NAD/NADH ratio under low oxygen level, because further increase of lactate concentration over 20 mM in medium was toxic to cells. Similar results were obtained using Bcap-37 cells (Figure S5).

The NAD/NADH ratio is a dependent variable responding to oxygen levels. Under hypoxia, the ratio is drastically reduced. Given the fact that NAD/NADH-involved oxidoreductive reactions are reversible and at near-equilibrium, the change of the ratio would likely alter the global biochemical processes in cells, i.e., the rates of many of these reactions would be significantly affected and many of the reactions would even proceed to the opposite direction under hypoxia, in comparison to those under normoxia. The change of biochemical processes corresponding to hypoxia-linked NAD/NADH, however, has not yet attracted sufficient attention.

It was noted that intracellular pyruvate was proportional to oxygen level, whereas lactate was negatively correlated with oxygen level (Figure 4E & F, Figure 5E & F). This might be explained by the mitochondrial shuttle, whose activity depends on oxygen level. Under normoxic condition, a fraction of NADH generated from glycolysis is recycled back to NAD via mitochondrial shuttle that spares a fraction of pyruvate. Under hypoxia, mitochondrial shuttle is blocked, and LDH catalyzed reaction is the only major way to turn NADH back to NAD, resulting in more pyruvate converting to lactate. As a result, less pyruvate and more lactate were observed under hypoxia than under normoxia.

In summary, our study (1) clarifies that NAD/NADH is stable and L/P is highly labile, that NAD/NADH is not a dependent variable responding to the change of L/P ratio, and that the highly labile L/P is crucial to maintain the stability of NAD/NADH; (2) reveals a long ignored blind spot in estimation of cytosolic free NAD/NADH ratio; (3) defines that the change of L/G and L/P could measure the status of conversion between pyruvate + NADH and lactate + NAD that tends to or gets away from equilibrium.

## Materials and Methods

### Measurement of Cell Division, Glucose Consumption, and Lactate Generation

Human breast cancer cell line Bcap-37, gastric cancer cell line SGC7901, and cervical carcinoma cell line Hela were maintained in RPMI-1640 (Invitrogen) with 10% FBS, 1% penicillin/streptomycin and 2 mM L-glutamine. Unless otherwise indicated, 8×10^5^ cells were seeded into six-well plate (Corning) to allow attachment overnight in a humidified CO2 incubator under normoxia or in a Workstation Invivo2 300 (RUSKINN) adjusted to a desired oxygen level (16%, 12%, 8%, 4%, or 1%). Then the culture medium was replaced with fresh RPMI-1640 medium supplemented with pure lactic acid to desired lactate concentration. At the indicated time, cell number, glucose consumption and lactate generation were determined. Cell count was carried out with a hematocytometer under optical microscope. Glucose was measured automatically by HK colorimetric method using Olympus AU2700 system. Lactate was determined by VITROS Chemistry Product LAC Slides using VITROS 5.1 FS system.

### Determination of Intracellular Pyruvate and Lactate

For intracellular pyruvate and lactate determination, cells in the plates were washed with ice-cold PBS 3 times and lysed with 500 µl acetonitrile, and transferred to eppendorf tube for centrifugation (20,000 g for 15 minutes at 4°C). 40 µl of supernatant was subjected for HPLC analysis for quantitative determination of intracellular pyruvate and lactate. An ICSEP ICE-ION-300 Column (Transgenomic) was attached to HEWLETT PACKARD series 1100 HPLC system and maintained at 57°C. The mobile phase was 0.005 M H2SO4 and the flow rate was 0.4 ml/min. The peak of UV trace (210 nm) corresponding to pyruvate (retention time, 15.3 min.) and lactate (retention time, 21.3 min.) were quantitated according to the standard curves.

### Calculation of NAD/NADH Ratio

The cytosolic lactate and pyruvate concentration were determined by HPLC as described above. NAD/NADH was estimated by the L/P ratio and the equation of the chemical equilibrium which is reported by Williamson et al [30]. Apparent Keq = [pyruvate]eq[NADH]eq[H+]/([Lactate]eq[NAD]eq) = 1.11×10^−11^, where pH is 7.0, If LDH catalyzed reaction is allowed to proceed to equilibrium, the final products and reactants could be expressed by the equation: Apparent Keq = Keq [H+], where Keq = [pyruvate]eq[NADH]eq/([Lactate]eq[NAD]eq) = 1.11×10^−7^ [48], [49], or [NAD]/[NADH] = [pyruvate]eq/(Keq[Lactate]eq).

## Supporting Information

Figure S1 **Intracellular lactate concentration and L/P ratio are highly labile in Hela cells.** Hela cells were incubated in complete RPMI-1640 medium containing 12 mM glucose and supplemented with 6 mM glucose every 24 hours. At the indicated time point, intracellular lactate and pyruvate, glucose consumption and lactate generation by Hela cells, and cell growth were monitored. (A) Intracellular lactate; (B) Intracellular pyruvate; (C) Intracellular L/P ratio; (D) Cytosolic free NAD/NADH ratio erroneously estimated from the corresponding L/P ratio; (E) Cell proliferation curves; (F) Glucose consumption; (G) Lactate generation; (H) L/G ratio (the generated lactate divided by the consumed glucose between 2 time points). Data are mean±SD. Data were confirmed by 2 independent experiments.
(DOC)

Figure S2 **Intracellular lactate concentration and L/P ratio are highly labile in SGC7901 cells.** SGC7901 cells were incubated in complete RPMI-1640 medium containing 12 mM glucose and supplemented with 6 mM glucose every 24 hours. At the indicated time point, intracellular lactate and pyruvate, glucose consumption and lactate generation by SGC7901 cells, and cell growth were monitored. (A) Intracellular lactate; (B) Intracellular pyruvate; (C) Intracellular L/P ratio; (D) Cytosolic free NAD/NADH ratio erroneously estimated from the corresponding L/P ratio; (E) Cell proliferation curves; (F) Glucose consumption; (G) Lactate generation; (H) L/G ratio (the generated lactate divided by the consumed glucose between 2 time points). Data are mean±SD. Data were confirmed by 2 independent experiments.
(DOC)

Figure S3 **Cytosolic free NAD/NADH ratio estimated at the conversion at or close to equilibrium in Hela cells.** Hela cells were incubated in complete RPMI-1640 medium containing 12 mM glucose supplemented with or without lactate. After 24-hour incubation, glucose consumption and lactate generation by Hela cells, cell growth, and intracellular lactate and pyruvate were measured. (A) Cell proliferation; (B) Glucose consumption; (C) Lactate generation; (D) L/G ratio; (E) Intracellular pyruvate; (F) Intracellular lactate; (G) Intracellular L/P ratio; (H) Cytosolic free NAD/NADH ratio (note that cytosolic free NAD/NADH at equilibrium of the conversion is 88.1±3.7) estimated from the corresponding L/P ratio. Data are mean±SD. Data were confirmed by 3 independent experiments.
(DOC)

Figure S4 **Cytosolic free NAD/NADH ratio estimated at the conversion at or close to equilibrium in SGC7901 cells.** SGC7901 cells were incubated in complete RPMI-1640 medium containing 12 mM glucose supplemented with or without lactate. After 24-hour incubation, glucose consumption and lactate generation by SGC7901 cells, cell growth, and intracellular lactate and pyruvate were measured. (A) Cell proliferation; (B) Glucose consumption; (C) Lactate generation; (D) L/G ratio; (E) Intracellular pyruvate; (F) Intracellular lactate; (G) Intracellular L/P ratio; (H) Cytosolic free NAD/NADH ratio (note that cytosolic free NAD/NADH at equilibrium of the conversion is 129.2±2.5) estimated from the corresponding L/P ratio. Data are mean±SD. Data were confirmed by 3 independent experiments.
(DOC)

Figure S5 **The effect of oxygen levels on cytosolic free NAD/NADH ratios.** Bcap-37 cells were incubated in complete RPMI-1640 medium containing 12 mM glucose supplemented with 20 mM lactate under 21% or 1% oxygen. After 24-hour incubation, glucose consumption and lactate generation by Bcap37 cells, cell growth, and intracellular lactate and pyruvate were measured. (A) Cell proliferation; (B) Glucose consumption; (C) Lactate generation; (D) L/G ratio; (E) Intracellular pyruvate; (F) Intracellular lactate; (G) Intracellular L/P ratio; (H) Cytosolic free NAD/NADH ratio estimated from the corresponding L/P ratio. Note that the NAD/NADH ratio under 1% oxygen should be far smaller than the value presented (see description in text). Data are mean±SD. Data were confirmed by 2 independent experiments.
(DOC)
