# Supplementary material for: Biochemical Issues in Estimation of Cytosolic Free NAD/NADH Ratio
Source: PLoS One. 2012 May 3;7(5):e34525. doi: 10.1371/journal.pone.0034525 (PMC3343042; doi:10.1371/journal.pone.0034525)
Supplement: Figure S2 — Intracellular lactate concentration and L/P ratio are highly labile in SGC7901 cells. SGC7901 cells were incubated in complete RPMI-1640 medium containing 12 mM glucose and supplemented with 6 mM glucose every 24 hours. At the indicated time point, intracellular lactate and pyruvate, glucose consumption and lactate generation by SGC7901 cells, and cell growth were monitored. (A) Intracellular lactate; (B) Intracellular pyruvate; (C) Intracellular L/P ratio; (D) Cytosolic free NAD/NADH ratio erroneously estimated from the corresponding L/P ratio; (E) Cell proliferation curves; (F) Glucose consumption; (G) Lactate generation; (H) L/G ratio (the generated lactate divided by the consumed glucose between 2 time points). Data are mean±SD. Data were confirmed by 2 independent experiments. (DOC) [file pone.0034525.s002.doc]

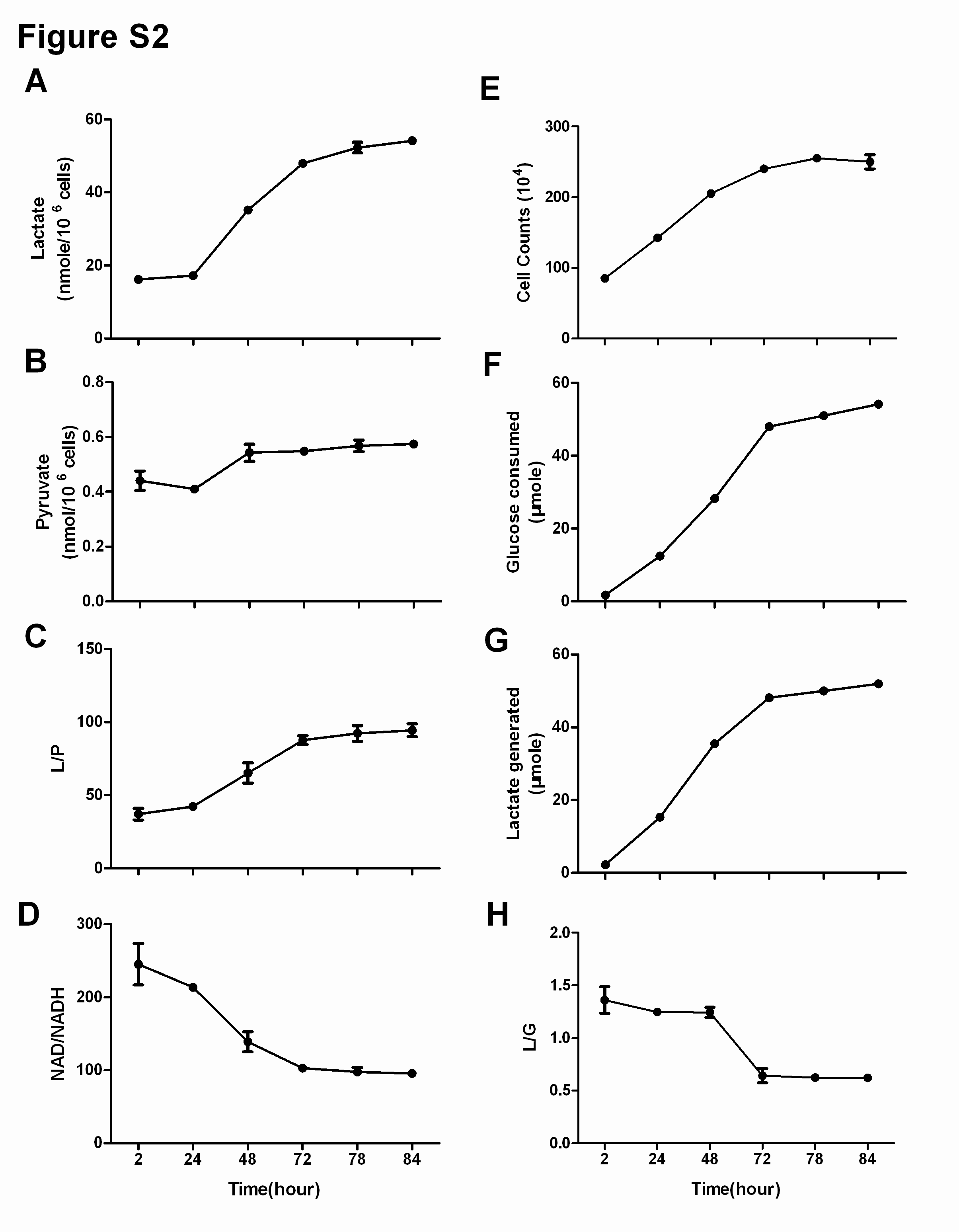
 Figure S2. Intracellular lactate concentration and L/P ratio are highly labile in SGC7901 cells. SGC7901 cells were incubated in complete RPMI-1640 medium containing 12 mM glucose and supplemented with 6 mM glucose every 24 hours. At the indicated time point, intracellular lactate and pyruvate, glucose consumption and lactate generation by SGC7901 cells, and cell growth were monitored. (A) Intracellular lactate; (B) Intracellular pyruvate; (C) Intracellular L/P ratio; (D) Cytosolic free NAD/NADH ratio erroneously estimated from the corresponding L/P ratio; (E) Cell proliferation curves; (F) Glucose consumption; (G) Lactate generation; (H) L/G ratio (the generated lactate divided by the consumed glucose between 2 time points). Data are mean ± SD. Data were confirmed by 2 independent experiments.
