# Supplementary material for: Biochemical Issues in Estimation of Cytosolic Free NAD/NADH Ratio
Source: PLoS One. 2012 May 3;7(5):e34525. doi: 10.1371/journal.pone.0034525 (PMC3343042; doi:10.1371/journal.pone.0034525)
Supplement: Figure S4 — Cytosolic free NAD/NADH ratio estimated at the conversion at or close to equilibrium in SGC7901 cells. SGC7901 cells were incubated in complete RPMI-1640 medium containing 12 mM glucose supplemented with or without lactate. After 24-hour incubation, glucose consumption and lactate generation by SGC7901 cells, cell growth, and intracellular lactate and pyruvate were measured. (A) Cell proliferation; (B) Glucose consumption; (C) Lactate generation; (D) L/G ratio; (E) Intracellular pyruvate; (F) Intracellular lactate; (G) Intracellular L/P ratio; (H) Cytosolic free NAD/NADH ratio (note that cytosolic free NAD/NADH at equilibrium of the conversion is 129.2±2.5) estimated from the corresponding L/P ratio. Data are mean±SD. Data were confirmed by 3 independent experiments. (DOC) [file pone.0034525.s004.doc]

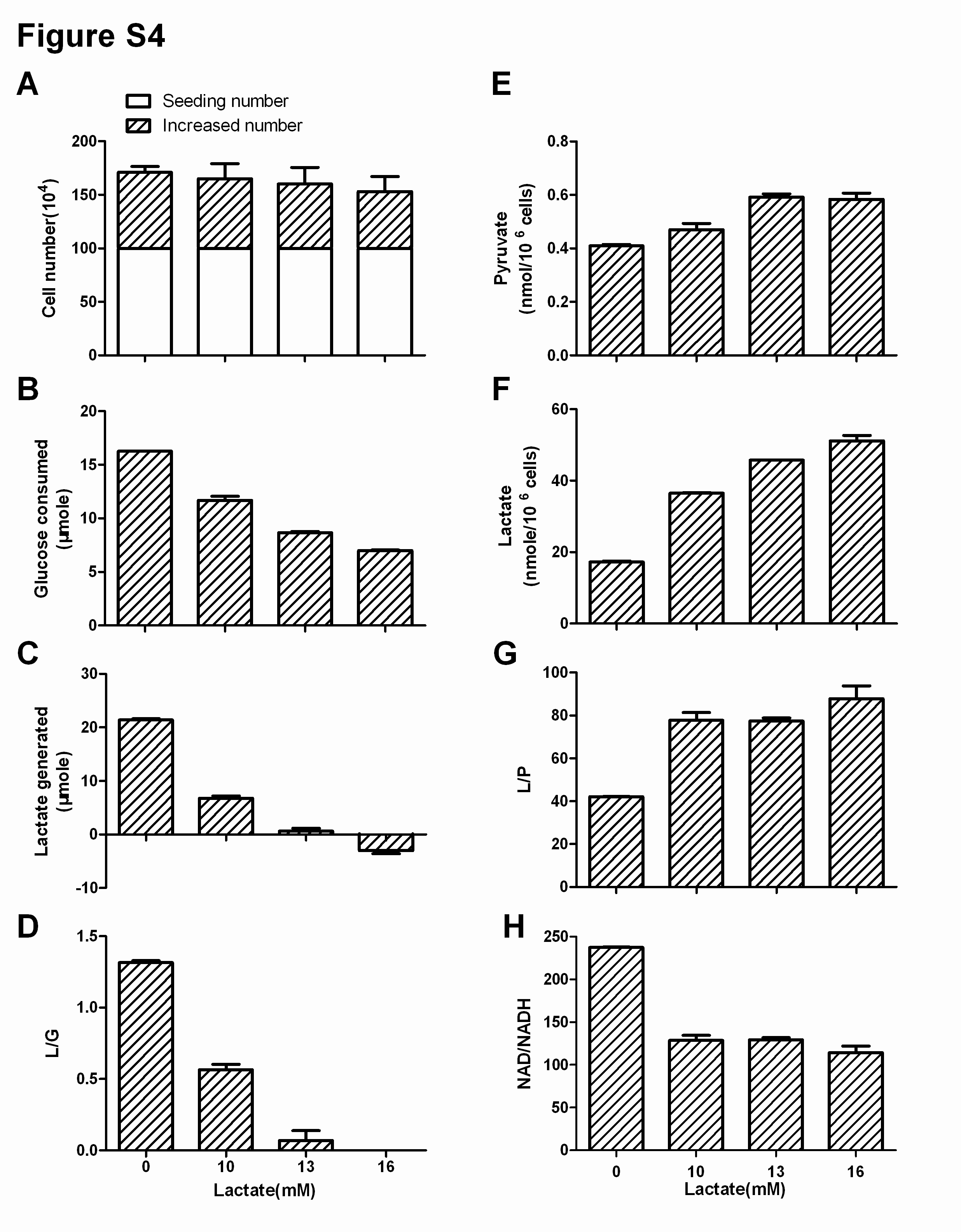


Figure S4. Cytosolic free NAD/NADH ratio estimated at the conversion at or close to equilibrium in SGC7901 cells. SGC7901 cells were incubated in complete RPMI-1640 medium containing 12 mM glucose supplemented with or without lactate. After 24-hour incubation, glucose consumption and lactate generation by SGC7901 cells, cell growth, and intracellular lactate and pyruvate were measured. (A) Cell proliferation; (B) Glucose consumption; (C) Lactate generation; (D) L/G ratio ; (E) Intracellular pyruvate; (F) Intracellular lactate; (G) Intracellular L/P ratio; (H) Cytosolic free NAD/NADH ratio (note that cytosolic free NAD/NADH at equilibrium of the conversion is 129.2 ± 2.5) estimated from the corresponding L/P ratio. Data are mean ± SD. Data were confirmed by 3 independent experiments.
