# Supplementary material for: Biochemical Issues in Estimation of Cytosolic Free NAD/NADH Ratio
Source: PLoS One. 2012 May 3;7(5):e34525. doi: 10.1371/journal.pone.0034525 (PMC3343042; doi:10.1371/journal.pone.0034525)
Supplement: Figure S5 — The effect of oxygen levels on cytosolic free NAD/NADH ratios. Bcap-37 cells were incubated in complete RPMI-1640 medium containing 12 mM glucose supplemented with 20 mM lactate under 21% or 1% oxygen. After 24-hour incubation, glucose consumption and lactate generation by Bcap37 cells, cell growth, and intracellular lactate and pyruvate were measured. (A) Cell proliferation; (B) Glucose consumption; (C) Lactate generation; (D) L/G ratio; (E) Intracellular pyruvate; (F) Intracellular lactate; (G) Intracellular L/P ratio; (H) Cytosolic free NAD/NADH ratio estimated from the corresponding L/P ratio. Note that the NAD/NADH ratio under 1% oxygen should be far smaller than the value presented (see description in text). Data are mean±SD. Data were confirmed by 2 independent experiments. (DOC) [file pone.0034525.s005.doc]

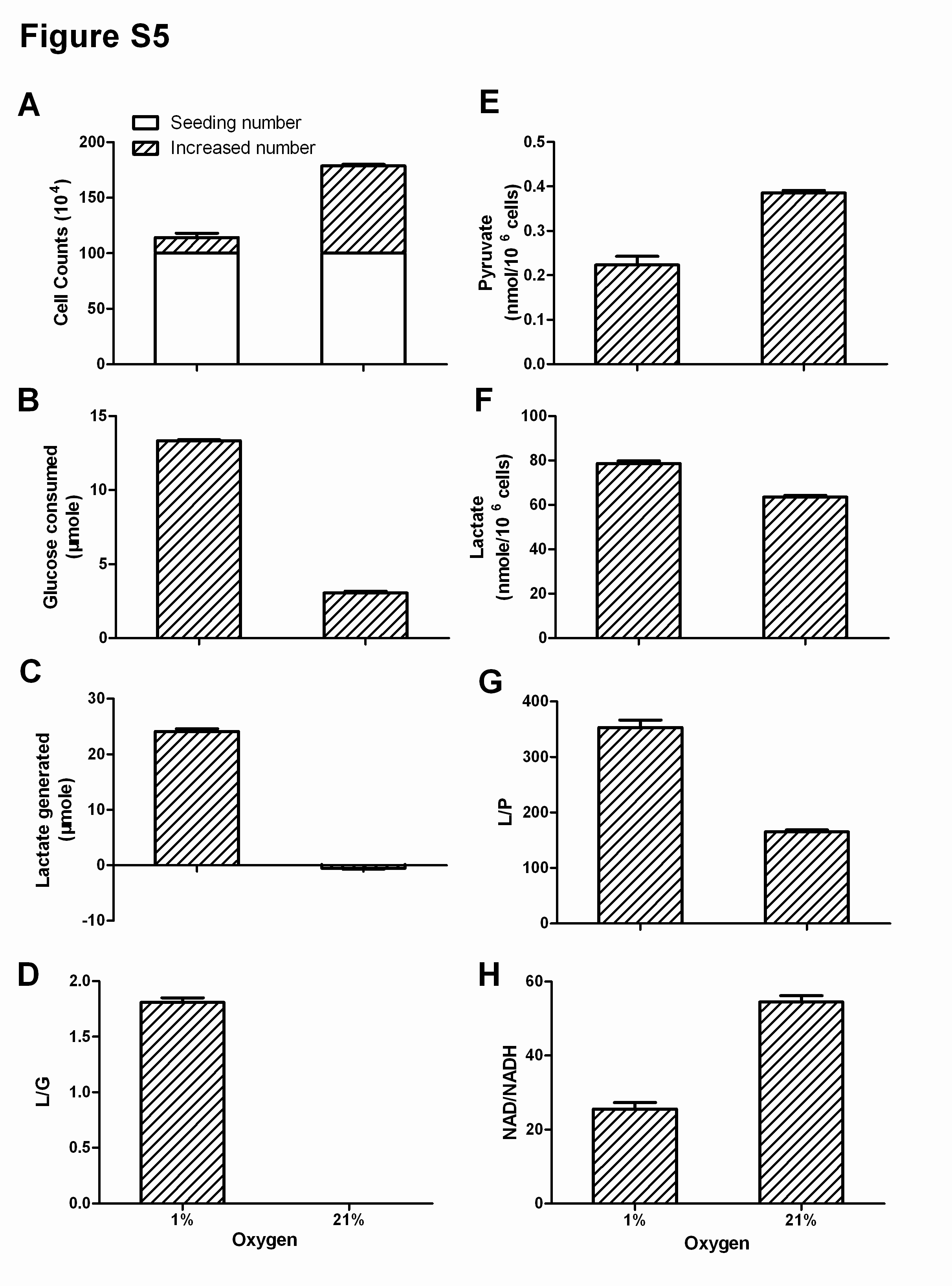


Figure S5. The effect of oxygen levels on cytosolic free NAD/NADH ratios. Bcap-37 cells were incubated in complete RPMI-1640 medium containing 12 mM glucose supplemented with 20 mM lactate under 21% or 1% oxygen. After 24-hour incubation, glucose consumption and lactate generation by Bcap37 cells, cell growth, and intracellular lactate and pyruvate were measured. (A) Cell proliferation; (B) Glucose consumption; (C) Lactate generation; (D) L/G ratio; (E) Intracellular pyruvate; (F) Intracellular lactate; (G) Intracellular L/P ratio; (H) Cytosolic free NAD/NADH ratio estimated from the corresponding L/P ratio. Note that the NAD/NADH ratio under 1% oxygen should be far smaller than the value presented (see description in text). Data are mean ± SD. Data were confirmed by 2 independent experiments.
